# A qualitative follow-up on perceptions of energy management education and daily life by individuals with fatigue undergoing hemodialysis treatment

**DOI:** 10.1177/03080226251335228

**Published:** 2025-05-30

**Authors:** Jane A Davis, Janine Farragher, Chandra Thomas, Pietro Ravani, Braden Manns, Brenda R Hemmelgarn

**Affiliations:** 1 Department of Occupational Science and Occupational Therapy, University of Toronto, Toronto, Canada; 2 Cumming School of Medicine, University of Calgary, Calgary, AB, Canada; 3 Faculty of Medicine and Dentistry, University of Alberta, Edmonton, AB, Canada

**Keywords:** Chronic kidney disease, occupational therapy, dialysis, life participation

## Abstract

**Introduction:**

People on chronic hemodialysis identify fatigue and its negative impact on their life participation (i.e., ability to accomplish valued daily occupations) as central to their illness experience. We explored how fatigue, as experienced by people on hemodialysis, shaped their participation in daily occupations, and participants’ experiences in the Personal Energy Planning programme.

**Method:**

This qualitative follow-up study to a pilot randomized controlled trial used an interpretive description approach with six participants who completed an energy management programme for people treated with hemodialysis in Calgary, Canada. Each participant completed one semistructured interview about their experiences with fatigue management, life participation and the programme. Data were analyzed using modified constant comparative analysis.

**Findings:**

Participants described moving from an illness experience to one of living, where they could envision participation in both activities of daily living and social leisure occupations. Participants also described how they felt supported by the programme with its focus on applying the learning from the programme to improve their participation.

**Conclusion:**

Fatigue has an extensive impact on various facets of life participation. For people treated with hemodialysis, the reconstruction of their occupational lives can be enabled through energy management education programmes focused on the development of personal metacognitive strategies.

## Introduction

Fatigue is one of the most common and debilitating complications experienced by people with kidney failure who undergo maintenance hemodialysis treatments. The symptom is so impactful that people on hemodialysis have recently chosen fatigue as one of four core health outcomes to measure in all clinical trials within nephrology. Unfortunately, the complex and multifactorial etiology of fatigue in people on hemodialysis has resulted in few treatment options that meaningfully reduce fatigue or its impacts in this population. Thus, we developed the Personal Energy Planning (PEP) programme, which applies the Cognitive Orientation to daily Occupational Performance (CO-OP) approach to help people on hemodialysis develop strategies to address their goals related to fatigue management and daily life. PEP was found to positively impact life participation among people on hemodialysis in a multisite, 1:1, pilot randomized controlled trial (RCT; Farragher et al., 2022), with participants demonstrating clinically significant improvements in meaningful activities. This article presents findings from a qualitative follow-up study to the pilot RCT, which explored how participants perceive fatigue as shaping their participation in daily occupations and their experiences participating in PEP.

### Literature review

People with kidney failure, who undergo hemodialysis experience multifactorial complications from their disease, comorbidities, and treatment regimen. They frequently live pain, nausea, and pruritis; frailty and muscular dysfunction; cognitive impairments; and depression and anxiety. Of these complications, the impact of fatigue on life participation was identified by people on hemodialysis as one of their top intervention priorities (Tong et al., 2017). Life participation is defined as the ability to perform and participate in valued day-to-day activities (Ju et al., 2018). The effects of fatigue on life participation in this population include an inability to fulfill relationship roles, frequent need to rest, reduced social participation, and reliance on others to support daily tasks (Clarkson, 2010; Lee et al., 2007; Jacobson et al., 2019; Ju et al., 2018; Rezaei et al., 2020). To date, a dearth of effective treatment options to address fatigue has left people on hemodialysis to manage this symptom largely on their own. Novel treatment approaches to mitigate fatigue and its impacts on everyday life are sorely needed.

The PEP programme is an energy management education programme developed specifically for people on chronic hemodialysis to improve their life participation. Energy management education approaches have been proposed to minimize fatigue and maximize life participation among people treated with hemodialysis. Energy management, also known as energy conservation or adaptive pacing, is an intervention recommended for use when fatigue interferes with daily life (Natale et al., 2023). It teaches people how to reduce their energy expenditure and increase their efficiency in occupational performance and life participation, using strategies such as prioritizing, organizing, delegating, and assistive equipment (Natale et al., 2023). PEP applies the CO-OP approach—an evidence-based, goal-focused, meta-cognitive, problem-solving approach—to guide patients in developing, considering, and applying energy management strategies that are feasible and tailored to their individual performance and participation goals. CO-OP has substantial evidence showing its positive effects on occupational performance and participation among people with diverse needs and challenges (see Dawson et al., 2017; https://www.icancoop.org/ for details about its theoretical basis and application). The PEP programme consists of 7–9 individualized weekly sessions that combine CO-OP with a self-guided, module-based component (Farragher et al., 2022). After completing three modules that introduce concepts and strategies relating to energy management, participants complete four to six 1:1 sessions with a facilitator trained in CO-OP and energy management. The facilitator uses specialized techniques from CO-OP to support patients’ problem-solving of their fatigue challenges, including guided discovery (a collaborative, problem-solving instruction method used to find solutions), dynamic performance analysis (DPA; an analytic observation-based process used to identify task performance problems), and “Goal-Plan-Do-Check” (a global metacognitive strategy for planning, implementing, and evaluating performance and participation outcomes; see Dawson et al., 2017).

In the pilot RCT conducted between July 2019 and December 2020, we found that the PEP programme was associated with meaningful improvements in occupational performance and/or participation for participants’ self-selected goals (Farragher et al., 2022). The current study extends what is known about fatigue and life participation for people on hemodialysis and their perceptions of the PEP programme by exploring the following research questions: (a) “How do people on hemodialysis perceive fatigue as shaping their occupational performance and participation?” and (b) “What are the participants’ experiences of the PEP energy management programme?”

## Method

### Study design

For this study, we used an interpretive description approach (Thompson Burdine at al., 2021; Thorne, 2016), which is a flexible research approach that supports the use of different strategies and analytic methods to address the specifics needs of a practice-based research question. In this instance, the approach, although not explicitly linked, was applied within a social constructionist paradigm (Holstein and Gubrium, 2011), recognizing the complexity and diversity of people’s experiences which are embedded within a societal context that shapes how people make sense of their daily lives. As such, we view the understandings generated from this study as subjectively experienced and can only ever be partially known. The study was approved by the Conjoined Health Research Ethics Board at the University of Calgary (#18-1657).

### Participants and recruitment

Participants were recruited from the 15 assigned to the pilot RCT intervention arm (Farragher et al., 2022) in Calgary, Canada who completed the programme. After the programme, all participants were approached in the hemodialysis unit by a postdoctoral fellow leading the study, who invited the participants to complete one qualitative follow-up interview. Six of 15 participants agreed to participate in the interview and provided informed written consent.

### Data collection

Within 2 weeks of their final PEP session, participants completed one semistructured interview, lasting 20–50 minutes. The interview guide (see Table 1) contained questions about participants’ experiences of fatigue within their daily lives before and after involvement in the programme, as well as the programme content and design. The interview was conducted in person or over the phone by the postdoctoral fellow, who has training in and experience with applying qualitative methodologies and facilitating interviews. All interviews were audio recorded.

**Table 1.** Interview guide.

Rationale	Question/Probe
Understanding fatigue	• Can you tell me about your experiences with fatigue prior to participating in this study?/In what ways was it affecting your life?
	• What did you do to manage fatigue before you participated in this study?
Trial acceptability Barriers to participating	• What were your initial thoughts or reactions when you were first told about the PEP fatigue study?
Intervention acceptability Mechanisms of action Factors affecting success Implementation of intervention in real world Fidelity/dose of intervention	• Now I’ll ask a few questions about the PEP program itself. Just to refresh your memory . . . the purpose of the PEP program was to help you learn how to use energy budgeting and energy-saving strategies to help manage your fatigue. • Can you share your thoughts about the PEP fatigue program after having participated in it? For example, things you liked or thought worked well about the program, and things you didn’t like or didn’t think worked so well about the program?
	○ What did you think of the PEP program’s approach of looking for ways to conserve energy in your daily life to manage fatigue?
	○ We designed three computer modules to teach you the basics about budgeting energy and saving energy. What feedback do you have about those modules?
	○ Tell me about the goals you set to work on for the PEP program, and a bit of history around why those three particular goals were important for you?
	○ We used a technique of having you imagine yourself doing your goal activities in detail to pinpoint specific ways that you could save energy. How well do you think that process worked?
	○ What were some of the energy-saving strategies that you came up with during the PEP sessions, and how did you find those strategies worked for you?
	○ We also used the problem-solving strategy of Goal-Plan-Do-Check to help guide the process of working on your fatigue goals. What did you think of this method of problem-solving?
	○ Did you have any other feedback on your sessions with [facilitator name]?
Perceived value and benefit of intervention	• In sum, can you describe how the PEP program has changed your experience of fatigue in daily life (if at all)? • Did this study live up to the expectations you had when you agreed to participate?
Final thoughts	• Is there anything beyond what we’ve talked about that we could have changed about the study or the PEP program that would have made it a better experience?
	• Is there anything else you would like to share about the PEP program study, or the PEP program, before we finish?

### Data analysis

Participant recordings were transcribed verbatim in Microsoft Word and analyzed using modified constant comparative analysis as described by Rennie (2006) and Charmaz (2017). This analytic process aligns with interpretive description and has been used outside of grounded theory (see Fram, 2013, for examples). Two researchers, both with experience in qualitative research—the postdoctoral fellow and an occupational therapist with no involvement in the pilot RCT—conducted the analysis.

Following data immersion, the two researchers independently applied an initial category to all self-identified meaning units of data in the transcripts (Rennie, 2006). Then, the two researchers met to compare their identified units and categories, one-by-one, grouping similar data units together to collapse initial categories into sub-themes (Charmaz, 2006; Rennie, 2006). Concurrently, three dominant concepts were identified by the researchers from the categorization process, generating a higher-order thematic structure for the analysis of the illness experience; the living experience; and the programme experience. The subthemes were then refined, named, and collated under the higher-order themes by both researchers to ensure consistency within and across the themes (see Table 2, for examples).

**Table 2.** Thematic structure and examples of analysis process.

Example meaning unit/quotation	Example initial category	Final theme	Final subtheme
“You think you are done but it’s trying to get back to your normal life is the hardest thing that I’m in a state right now just because I can’t do all those things anymore. I cannot be that quick. I cannot not be, there’s a lot that slowed me down.”	Frustration with life on hold	Illness experience	Putting life on hold: Navigating the uncertainty of fatigue
“No energy to cook, no energy to do anything because I’d like to say, I like to use whatever hours I have after work to just rest, I don’t want to move anymore, I don’t want to do anything.”	Tired of being tired		
“I guess very regular because one day I got very sick and another day, ok. I mean, very difficult to predict how things will be”	Fatigue uncertainty		
“Yeah it [fatigue] was really quite bad. Dialysis days were a complete, like today, complete write-off for me. I finish dialysis, go home, lay down, sleep for probably four hours.”	Putting life on hold		
“Because you are asking a favour, as much as they, they are very supportive, but I just feel bad because I’m not like this.”	Feeling dependent on others		Moving toward living: Managing feelings of dependence and guilt
“I feel guilty because my mom is not that young, she’s my dietician sort of. I don’t know, it’s juggling work and home and dialysis.”	Feelings of guilt from needing support		
“They modified my work, he modified my work, you know the guilt is stressing me.”	Managing guilt while trying to move back to living		
“I like to do something when I’m at home and being alone you get bored right . . . I will sort my laundry, I take my time and then from the second floor I drop it on the floor. So I don’t have to carry the whole load, but it’s extra step but a lighter step that I can do and then I rest in between.”	Routine compensation	Living experience	Making simple difficult and difficult simple
“I walk my dog at least once or twice a week. Sometimes three times is too much, so I don’t do it three times.”	Habit modification		Restructuring daily occupations and routines
“I do get my mom to help me [with the laundry] on days where I feel like I can’t do it all myself”	Asking for help		Outsourcing and seeking support
“[I don’t have] the energy to do the ordinary things . . . but if somebody invited me out for lunch I could go”	Occupational prioritization		Planning and scheduling the day-to-day
“Talking it over with them and talking over the stats . . . helped me organize my thoughts.”	Facilitator support	The programme experience	Feeling supported and understood
“Reading is one thing because I like to read the module, but [by] discussing it you get more ideas from the person that’s assigned with the program.”	Expanding ideas and trying it out		Applying the learning beyond talk and text
I don’t know how much I changed [because of PEP] because a lot of it I was doing already because when you are tired you tend to find a way around things.	Limited new possibilities		Envisioning new daily life possibilities
“[the PEP program] opened up a lot of options too. You are not restricted to what you normally do just because you can’t do it, you don’t do it at all. It’s been an excellent, it’s an excellent program I should say and I discovered that, oh, I didn’t realize that, I thought it was just ok to stick with the same routine or same, you know how to say it, I don’t know how to say it.”	Expanding the perception of possibilities		

### Trustworthiness

Credibility of the study’s findings was maximized by applying the following strategies. The interviews were conducted by a postdoctoral fellow who was unknown to the study participants and not involved in administering the study intervention. The interview guide was co-developed by the postdoctoral fellow and reviewed by research team members and a patient partner to reduce bias and maximize its relevance to the population. The data were analyzed by two researchers, one of whom had no prior involvement with PEP programme development or research, which allowed for triangulation of researcher interpretation supporting the dependability of the analysis.

## Findings

### Participant characteristics

Three participants identified as male and three as female, with a mean age of 53.7 years (range = 21–67 years). Their median dialysis vintage was 3.7 years (range = 1.7–8.6 years), 3 of 6 had chronic kidney disease + diabetes, and 2 of 6 participants scored above the established cutoff for depressed mood (⩾2) on the Patient Health Questionnaire (PHQ) 2.

### Qualitative findings

This study explored how people on hemodialysis perceive fatigue as shaping their occupational performance and participation and their experiences of the PEP programme. Three themes were generated from the data, each comprising subthemes that characterized the participants’ journey alongside the programme (see Table 2). The six participants are identified using pseudonyms to protect their anonymity.

### The illness experience

When asked to reflect on their experiences of fatigue prior to participating in the programme, participants often described these experiences in relation to days on and off dialysis; however, for many, the constant fatigue overwhelmed their lives. Their illness experience was conveyed as an iterative process from participants’ initial experiences of their illness and dialysis to restructuring their thoughts and lives through two subthemes: (a) Putting life on hold: Navigating the uncertainty of fatigue; and (b) Moving toward living: Managing feelings of dependence and guilt.

#### Putting life on hold: Navigating the uncertainty of fatigue

Participants spoke about the uncertainty of each day and their future because of their illness, especially because of their dialysis and associated fatigue.

It’s hard for me to be like, I can do this on that day. Because I wake up tomorrow . . . and I can’t get out of bed. I feel too sick . . . It’s hard for me to be able to think about things in the long run. (Gemma)

Fatigue was perceived by participants as the most all-encompassing part of their illness experience: “I’d go to sleep fatigued and wake up fatigued” (Ming). Participants were unable to plan beyond the moment, putting their lives on hold: “I have no life . . . it’s mostly sleeping now . . . there’s not really anything you can do” (Roland).

Participants conveyed how simply planning for the day-to-day led to fatigue: “It’s very hard. The fatigue of thinking, the exhaustion of thinking, ok who’s going to drive me?” (Aaliyah). Some participants reported specifically about dialysis days and how that impacted their typical routines, such as work: “I could not work on dialysis days” (Ming). For one participant who was able to continue working, the fatigue meant that she lacked the energy to do anything else: “I have no energy to cook; no energy to do anything because I like to use whatever hours I have after work to just rest” (Aaliyah). Rest and sleep were viewed as the only occupations that would help manage fatigue.

#### Moving toward living: Managing feelings of dependence and guilt

Participants spoke about needing to start rebuilding their lives despite the fatigue but did not know how: “Trying to get back to your normal life is the hardest thing. I’m in a state right now because I can’t do all those things anymore. I cannot be that quick, there’s a lot that slowed me down” (Aaliyah). Daily routines were interrupted by fatigue, which led to feelings of panic: “You have not accomplished anything. Sometimes it panics me, I say, ok what am I going to do next. Ok I have to get calm and then you plan” (Aaliyah). Participants conveyed their desire to regain their sense of self and participate in daily life:

My spasms are bad today. I’m so tired, but I wanted to clean better. I wanted to be able to do my laundry myself because my mom helps me a lot. . . . It’s a struggle with my fatigue to do it [clean] as often and to the standards that I want. (Gemma)

Participants spoke about their dependence on help from others to make it through, especially, dialysis days. One participant spoke of the guilt from requesting work accommodations: “He modified my work; you know the guilt is stressing me” (Aaliyah).

### The living experience

All participants spoke about working to rebuild their occupational lives. Some discussed existing strategies, while others commented on how their involvement in the programme helped them to figure out new or refine current ways of doing to maximize their participation. Four subthemes were identified: (a) making simple difficult and difficult simple; (b) restructuring daily occupations and routines; (c) outsourcing and seeking support; and (d) planning and scheduling the day-to-day.

#### Making simple difficult and difficult simple

Participants spoke about how fatigue and dialysis made typically simple activities difficult, leading them to find ways of simplifying their performance.

I said to myself, ok when am I tired? Ok, well I’m tired after I do the shower and the shave together. I was tired when I was putting on a suit. I was tired; I never used to eat at my desk at the office. (Ming)

As Lisa stated, “day-to-day things [now] take twice as long to do.” Trying to do what they used to do required extra time and increased effort: “You did everything you had to do—cooking and the cleaning—but it was always an effort. It takes longer” (Arjun). Participants conveyed how because of fatigue, many tasks were left undone: “I have no energy, and you know, I’ll start something and then I can’t finish it . . . I look at dishes . . . sitting in the sink” (Lisa). Aaliyah’s family had offered solutions, but the extra time and effort required just to make it through the day stopped her from using them: “My kids bought me three stools, but I didn’t even have time to think of taking out the stool.” Regardless of the extra time and effort it took, participants wanted “the energy to do the ordinary things” (Lisa).

Simplification of difficult daily occupations was a primary focus for participants to support participation in daily life. Participants discovered that breaking difficult occupations down into smaller tasks and doing “practical things [made] a difference” (Lisa). As one participant stated, “Why would I force myself to do it all at once in one day” (Aaliyah). Yet, for some occupations, when simplified, the number of discrete tasks grew:

You plan your goal, you think, rethink and execute it . . . you have to modify things. It would be 10 steps longer, but it saves you 10 steps from being fatigued . . . it’s stretching the time but minimizing the energy you use, resting in between. (Aaliyah)

Participants found that they were able to perform more difficult occupations by making small changes that added up: “a bunch of minor stuff, minor changes, when you put them altogether, I think helps. Well, I know it helped” (Ming).

#### Restructuring daily occupations and routines

Participants conveyed specific strategies for restructuring their routines, adapting their occupations, and prioritizing what was important for them to do with their energy. On dialysis days, participants used sleep as a strategy: “When I come home from dialysis, I have a nap. I try not to nap on other days” (Lisa). Other participants restructured their routines to spread an occupation over multiple days:

I started ironing my husband’s clothes . . . normally I do dozens of them, and now, why would I tire myself doing two dozen shirts at once when I can do five only at a time or leave [the rest] for the next day. (Aaliyah)

Similarly, Roland described how he breaks up his yardwork into smaller tasks: “We said that maybe I can do some of it. When I get tired, I’ll stop and go rest. Do a little bit at a time instead of doing the whole thing [in one day].” Participants would also restructure their routines by doing an occupation less frequently: “I walk my dog at least once or twice a week. Sometimes three times is too much” (Gemma). Restructuring their schedule of one occupation afforded participants the ability to participate in others: “Working 5 and a half, 6 hours a day [less hours over more days] has made a huge difference. I can now come home from work and do something.” (Ming). Another participant spoke of how she restructured several occupations across multiple days to help with fatigue: “I’ll do my bed one day and then I’ll start doing the floors [another day], sweeping and stuff, then I’ll organize what needs to be organized and I’ll put my clothes away (Gemma).”

Participants used various discrete strategies to adapt the form of their daily occupations. One participant described how she now gathered things in one place, especially when gardening: “She [the facilitator] said keep your [gardening] tools all in one place so that you are not looking for them” (Arjun). Another participant reported the impact of sitting to do her occupations: “The stool is my favorite [strategy] . . . it answered a lot of my problems” (Aaliyah). A third participant discussed buying different types of clothing to ease dressing: “We talked about helping myself to get dressed because I have very sore shoulders and I find that hard to do . . . so getting blouses that button up rather than pulling a t-shirt over my head” (Lisa) and using “the picker-upper [long handled reacher] when I need to” (Lisa). Laundry was a common household chore that participants found very exhausting: “From the washer to the dryer, that’s the hardest thing for me” (Aailyah). Thus, participants often adapted how they performed laundry: “My usual strategy is taking a pile [of clothes] and throw[ing] them down the stairs. Then going downstairs to do my laundry so that I don’t have to keep going up and down” (Gemma).

Participants spoke about prioritizing their well-being and their valued occupations over doing “mandatory tasks” that could be left for later or done by someone else: “I don’t have to overwhelm myself. I know it will get done; it’s just a matter of how long or how fast I want to get [it] done” (Aaliyah). Prioritizing not only meant changing what participants viewed as priorities but also challenging internalized expectations about whether and how things must be done. Some participants prioritized social events that impacted their psychological well-being: “[I don’t have] the energy to do the ordinary things . . . but if somebody invited me out for lunch I could go” (Lisa).

#### Outsourcing and seeking support

Participants spoke of having others help with or complete daily occupations for them: “I do get my mom to help me [with the laundry] on days where I feel like I can’t do it all myself” (Gemma). Outsourcing the work to others who were willing and able to support the participants, especially on dialysis days was crucial: “My husband is helping me with [chores] right now” and “my mom cooks for me [on dialysis days]” (Aaliyah). One participant moved to a seniors’ residence “because they do all the cooking and cleaning, all that kind of stuff” (Ming). Another participant found support from her boss:

At work [I asked my boss] if I could take a break every hour, or every two hours, a 15-minute break [to] regain my energy and he said, “you can have a nap.” And because I can’t sleep, he bought me a couch. (Aaliyah)

A participant who lived alone did not have the same options for support but wished she did: “I look at dishes . . . sitting in the sink and I think, oh well . . . maybe the fairy will come and do them” (Lisa).

#### Planning and scheduling the day-to-day

To maintain their daily routines and occupational repertoire, participants had to plan and schedule their day-to-day to ensure that everything was organized so they could do the things they wanted to do. Their planning and scheduling on dialysis days often involved coordinating the help of family and friends:

The fatigue of thinking, the exhaustion of thinking, who’s going to drive me? . . . the day before I have to ask my brother, my sister, and my brother-in-law [to take me to dialysis] and then my kids will pick me up in the evening after dialysis. I have to schedule that all the time on top of what I’m trying to schedule. (Aaliyah)

Participants discussed how organizing their days was tiring but important to ensure that they had social time: “I can think, I can plan, like strategize how to deal with working at the same time and being with my family. It’s tough” (Aaliyah). One participant noted how she used to only do social outings on specific days but with her new strategy, she can now do social things on other days: “I was very much saying to myself, ok, Sunday afternoon, Monday are the only days I can [plan to go out], [but now] I have a date Wednesday night for example” (Gemma). Participants also describe the smaller plans that were needed to carry out their daily occupations: “If I need a bowl down [from the cupboard] in the morning, I’ll try and remember to ask my husband to take it out the night before. Just planning ahead” (Aaliyah).

### The programme experience

The programme experience describes participants’ perceptions of PEP that facilitated changes from their illness to living experiences. Three subthemes captured their programme experience: (a) Feeling supported and understood; (b) Applying the learning beyond talk and text; and (c) Envisioning new daily life possibilities.

#### Feeling supported and understood

Participants spoke about the support they received from the facilitator, as it helped to reinforce participants’ ideas: “Talking it over with them and talking over the stats . . . helped me organize my thoughts. Was I simplifying it enough? Or did I miss something that I could have done to make things easier?” (Gemma). Consequently, participants’ programme involvement led to receiving more support and understanding from family members: “Whatever I learned from [the facilitator] I share with my husband, [so that] . . . he’s more aware of the things and then he helps me. I wasn’t feeling well but he says, do you want to plan for tomorrow?” (Gemma). Other participants stated that they wanted to figure out their own strategies: “I know I have all the other support that’s around me, but I have to do something for myself. I said, they can suggest things, but it has to come from me” (Aaliyah).

#### Applying the learning beyond talk and text

The facilitators were perceived to help participants apply the learning beyond talk and text: “Reading is one thing because I like to read the module, but [by] discussing it you get more ideas from the person that’s assigned with the program” (Aaliyah). This application was important as participants noted that they were already talking about their fatigue: “What else can you do with fatigue by just talking about it, right? You could take drugs to lower your fatigue, but I mean that’s not what I want to do” (Roland). Facilitators were perceived as a guide who helped participants uncover ideas to help manage their fatigue: “It is really helpful for anyone . . . [to have] someone to guide them on how to deal with the ideas, shortcuts just to deal with fatigue” (Aaliyah).

Participants spoke about tools that helped with the application of what they learned, such as DPA: “[The visualization DPA was helpful] because I broke my day down. So you get up and then come up with some little things around what I do every day” (Ming), and global strategies like Goal, Plan, Do, Check: “It makes a big difference, actually that tool is part of why, I’m trying to get back to work” (Aaliyah).

#### Envisioning new daily life possibilities

Participants discussed envisioning new possibilities for participation following their involvement in the programme. One participant spoke of how the programme helped her focus on specific and meaningful goals: “They were so personal. Sometimes the goals can be very vague? You know, I will try and do such and such, but this was really specific to me” (Lisa). PEP’s individualized approach enabled participants to focus on changing small everyday aspects of their life: “I discovered things that I didn’t know that I should be doing, or I didn’t know I could have done. It gives you more information [about] other ways to get things done easier, faster, and by not exhausting yourself” (Aaliyah).

Other participants directly related their PEP involvement to rebuilding their social lives beyond work: “The big one [how the programme has changed my life] is I can now come home from work and do something. You know, if the guys phone and they want to go have a beer, or even go on a date, I now have the energy” (Ming). Overall, most participants found that the programme increased possibilities for managing their fatigue to support greater participation in their daily lives broadly: “[The programme] has made me more aware of how I can change it [my life]” (Lisa). It helped participants gain metacognitive awareness and strategies through understanding that they did not have to stay doing their previous exhausting routines:

It [the programme] opened up a lot of options. You are not restricted to what you normally do because you can’t do it. It’s been an excellent program . . . I thought it was just ok to stick with the same routine or same [way of doing things]. (Aaliyah)

Although participants reported benefits from PEP, the impact of perceived benefits was tempered: “We went through some ideas, and I tried those ideas, and you know, they seemed to be what I would do before anyways” (Roland). Arjun also questioned how much the programme impacted his participation: “I don’t know how much I changed [because of the programme] because a lot of it I was doing already because when you are tired you tend to find a way around things” (Arjun); however, Arjun also found that the programme helped him to find “better ways of doing things” that he wanted or needed to do. Another participant captured some of this nuanced impact that the programme has on metacognition: “I thought the program got me thinking about stuff that, you know, I would have never thought of” (Ming). One participant, who had previously engaged in energy management programmes, did not feel that the programme offered them anything new:

I try my best to continue with my steps and to continue getting stronger, but I don’t [get stronger]. It didn’t make that much of a difference, but I already had my own thing [to manage fatigue because of my previous diagnosis]. So, I had a hard time making new schedules and stuff like that when I am doing the step-by-step thing . . . I don’t know how to simplify it [laundry] anymore and she was like “what can we do to make it easier?” And, it was like, I don’t know. I just felt like kind of a waste of time because I didn’t know any more ways to simplify the steps. (Gemma)

## Discussion and implications

This study explored how fatigue, as experienced by people on hemodialysis, shaped their occupational performance and life participation, and their perceptions of the PEP programme. Participants in our study experienced significant fatigue at their illness outset and during hemodialysis, which impacted their occupational routines. These findings align with those from a study by Rani and Kalia (2022), who found that 70% of patients undergoing hemodialysis (*n* = 100) reported moderate or severe fatigue with 47% requiring moderate or full support for the performance of activities of daily living. Brys et al. (2021) similarly examined the activity patterns of 42 participants from a hemodialysis unit in the Netherlands and found 48% of participants’ daily activity patterns comprised passive occupations (resting, watching TV, doing nothing, and relaxing). A smaller study by Farragher et al. (2024) found that the most common occupational performance problems reported by people treated with dialysis who experienced fatigue were household management and self-care. Our study provided additional clarity regarding how some people on dialysis experience their activity patterns, as our participants spoke of their difficulty in navigating the uncertainty or inconsistency of their fatigue and its impact on their daily occupations, leading to experiences of shame and guilt due to the need for family support.

The themes of illness experience and living experience illustrate the participants’ process of occupational change from managing their symptoms of kidney disease and hemodialysis to beginning to rebuild their lives. In a classical paper, Bury (1982) applied the term “biographical disruption” to illustrate the process of identity disruption due to chronic illness, and the corresponding term, “biographical repair,” which focuses on learning and using strategies to cope with one’s illness. In their thematic synthesis of qualitative studies, Elias et al. (2025) applied Bury’s framework to people living with late-stage kidney disease, highlighting this same process of disruption and repair across multiple studies. Although Elias et al. (2025) alluded to the concept of occupational functioning, such as performing household chores, their concept of biographical repair was predominantly related to coping with illness. Our findings highlight how people on hemodialysis want to experience living again, moving beyond coping with their illness and fatigue. Participants conveyed how they wanted to rebuild their occupational repertoires by rescheduling their occupations at times when they have more energy and making often simple, yet significant, changes to the form and performance of their occupations. Nelson (1988) introduced the term “occupational form” to capture the specific structure and organization of people’s daily occupations, including the ways they are performed and subsequent routines that emerge (Nelson, 1988). The participants in our study were encouraged to examine the tasks, materials, procedures, and contexts of their occupations to uncover new ways of doing, which increased their occupational engagement and participation. Interestingly, Kuluski et al. (2014) noted similar focus on living, characterizing it as “life regained,” for young individuals living with chronic stroke. In contrast to Elias et al. (2025), our findings pertaining to the living experience may be due to the societal assumption that people with life-limiting conditions cannot or are not interested in rebuilding their lives or “restoring normality” (Kuluski et al., 2014) but instead remain focused on coping with their illness. Moreels et al. (2023) reported a similar adaptation process experienced by 12 study participants with kidney failure, but did not overlay the process of adaptation with an energy management approach; thus, they presented potential areas for remediation including the social environment; developing new daily activity patterns; and meaningful occupations and goals. These findings highlight the potential of occupation-based interventions for this population to develop strategies for meaningful occupational engagement and continued life participation in the context of life-limiting illness.

The findings of our study have direct relevance to the Royal College of Occupational Therapists’ top ten priorities for occupational therapy research in the United Kingdom (https://www.rcot.co.uk/top-10). First, they provide insight into how occupational therapy can provide support for the occupational lives of people with kidney failure. Participants discussed how they could envision new possibilities for their life participation following their involvement in PEP. They spoke of the benefits of working on personalized goals that were meaningful to them, and the value of receiving support with identifying fatigue management strategies they could implement in their daily lives. These findings are important because they highlight the potential contributions of occupational therapy to supporting people with kidney failure. A recent study found that access to occupational therapy within this population is currently limited in the United Kingdom, with only 7% of dialysis units providing outpatient kidney-specific rehabilitation services and just 9% offering outpatient support to facilitate meaningful activity (Ancliffe et al., 2024). Advocacy from the occupational therapy profession remains vital for people with kidney disease. Our findings also illustrate how occupational therapy can support self-management. The use of CO-OP within our energy management program was intended to promote self-management of fatigue via techniques, such as guided discovery, Goal-Plan-Do-Check, and DPA, which have been shown to enable self-navigation of occupational performance problems in other populations (Dawson et al., 2017). In our study, participants with kidney failure similarly spoke about how these techniques were helpful in facilitating their application of energy management strategies to promote their life participation. Our findings suggest that the PEP programme may promote strategy development among people on hemodialysis with fatigue to improve areas such as social participation and the planning/scheduling of occupations, leading to new activity patterns and improved participation in meaningful and purposeful occupations.

### Implications for practice and future research

This study underscores the importance of providing dedicated support to people on hemodialysis with participating in valued occupations. At present, evidence-based interventions for promoting occupational participation in this population are scarce (Ancliffe et al., 2024). Our findings suggest that programs like the programme have the potential to make a meaningful difference in the quality of life of this group of patients. Randomized controlled trials that robustly test the efficacy of the programme and similar programs for supporting life participation for people living with chronic kidney disease are a priority for future research.

### Strengths and limitations of study

One strength of the study is that two researchers, one external to the study, completed the analytic process independently. This process helped to mitigate biases in the analysis process. We did not use member checking as part of this study as the timing of our analysis did not afford the opportunity. We attempted to remain as close to the data as possible with our analysis, while also providing meaningful interpretations of the data that would address our practice-based research question. We also acknowledge the limited participant demographic information provided, which will impact transferability of our findings by practitioners. Finally, our sample size was limited by the number of participants in the pilot RCT. Although we did approach data saturation with our analysis, having one or two more participants may have helped to deepen our analysis of a couple of less-developed subthemes.

## Conclusion

Fatigue has an extensive impact on various facets of life participation in people treated with hemodialysis and often requires occupational restructuring. Moving from an illness experience to one of living, where people can envision participation in both activities of daily living and social/leisure occupations, may be enabled through energy management education programmes focused on development of personal metacognitive strategies.

**Key findings**

- Fatigue impact is pervasive and requires a restructuring of occupations and routines, as well as changes in occupational form for people treated with hemodialysis.
- Energy management programmes using metacognitive strategy training may positively impact occupational participation.

**What the study has added**

Energy management education programmes may create positive impacts on the living experience, including changes in occupational form, routines, and participation, for people treated with hemodialysis and living with fatigue.

## References

[bibr1-03080226251335228] Ancliffe L Castle EM Wilkinson TJ , et al. (2024) A national survey of current rehabilitation service provisions for people living with chronic kidney disease in the UK: Implications for policy and practice. BMC Nephrology 25: 302. 39266986 10.1186/s12882-024-03742-4 PMC11391674

[bibr2-03080226251335228] Brys ADH Stifft F Van Heugten CM , et al. (2021) mHealth-based experience sampling method to identify fatigue in the context of daily life in haemodialysis patients. Clinical Kidney Journal 14: 245–254. 33564425 10.1093/ckj/sfaa124 PMC7857808

[bibr3-03080226251335228] Bury M (1982) Chronic illness as biographical disruption. Sociology of Health and Illness 4: 167–182. 10260456 10.1111/1467-9566.ep11339939

[bibr4-03080226251335228] Charmaz K (2006) Constructing Grounded Theory: A Practical Guide Through Qualitative Analysis. Thousand Oaks, CA: Sage.

[bibr5-03080226251335228] Charmaz K (2017) Special invited paper: Continuities, contradictions, and critical inquiry in grounded theory. International Journal of Qualitative Methods 16: 1–8.

[bibr6-03080226251335228] Clarkson KA Robinson K (2010) Life on dialysis: A lived experience. Nephrology Nursing Journal 37: 29–35. 20333901

[bibr7-03080226251335228] Dawson DR McEwen SE Polatajko HJ (Eds) (2017) Cognitive Orientation to Daily Occupational Performance in Occupational Therapy. Using CO-OP Approach^TM^ to Enable Participation During Life Span. Bethesda: AOTA press.

[bibr8-03080226251335228] Elias MA Van Damme W Ku GMV , et al. (2025) Lived expériences of people with chronic kidney disease on maintenance dialysis: A systematic review and thematic synthesis of qualitative studies. BMC Nephrology 26: 22. 39806270 10.1186/s12882-025-03952-4 PMC11731171

[bibr9-03080226251335228] Farragher JF Ravani P Manns B , et al. (2022) A pilot randomised controlled trial of an energy management programme for adults on maintenance haemodialysis: The fatigue-HD study. BMJ Open 12. 10.1136/bmjopen-2021-051475 PMC8845206 35144947

[bibr10-03080226251335228] Farragher J Davis JA Thomas C , et al. (2024) Occupational priorities of people on hemodialysis who participated in energy management education. Canadian Journal of Occupational Therapy (online ahead of print). 10.1177/00084174241271205 PMC12117131 39113490

[bibr11-03080226251335228] Fram SM (2013) The constant comparative analysis method outside of grounded theory. The Qualitative Report, 18: 1–25.

[bibr12-03080226251335228] Holstein JA Gubrium JF (2011) The constructionist analytics of interpretive practice. In Denzin NK Lincoln YS (Eds.), The SAGE Handbook of Qualitative Research (pp. 341–357). Thousand Oaks: Sage.

[bibr13-03080226251335228] Jacobson J Ju A Baumgart A , et al. (2019) Patient perspectives on the meaning and impact of fatigue in hemodialysis: A systematic review and thematic analysis of qualitative studies. American Journal of Kidney Diseases 74: 179–192. 30955947 10.1053/j.ajkd.2019.01.034

[bibr14-03080226251335228] Ju A Unruh ML Davison SN , et al. (2018) Patient-reported outcome measures for fatigue in patients on hemodialysis: A systematic review. American Journal of Kidney Diseases 71: 327–343. 29198388 10.1053/j.ajkd.2017.08.019

[bibr15-03080226251335228] Kuluski K Dow C Locock L , et al. (2014) Life interrupted and life regained? Coping with stroke at a young age. International Journal of Qualitative Studies on Health and Well-Being 9: 22252. 10.3402/qhw.v9.22252 PMC3901846 24461569

[bibr16-03080226251335228] Lee B-O Lin C-C Chaboyer W , et al. (2007) The fatigue experience of haemodialysis patients in Taiwan. Journal of Clinical Nursing 16: 407–413. 17239077 10.1111/j.1365-2702.2005.01409.x

[bibr17-03080226251335228] Moreels T Van de Velde D Van Duyse S , et al. (2023) The impact of in-centre haemodialysis treatment on the everyday life of older adults with end-stage kidney disease: A qualitative study. Clinical Kidney Journal 16: 1674–1683. 37779844 10.1093/ckj/sfad104 PMC10539253

[bibr18-03080226251335228] Natale P Ju A Strippoli GF , et al (2023) Interventions for fatigue in people with kidney failure requiring dialysis. Cochrane Database of Systematic Reviews 8: CD013074. 10.1002/14651858.CD013074.pub2 PMC10468823 37651553

[bibr19-03080226251335228] Nelson D (1988) Occupation: Form and performance. American Journal of Occupational Therapy 42(10): 633–641. 10.5014/ajot.42.10.633 3059801

[bibr20-03080226251335228] Rani D Kalia R (2022) Activities of daily living (ADL) and fatigue among patients undergoing hemodialysis. Nursing and Midwifery Research Journal 18: 88–96.

[bibr21-03080226251335228] Rennie DL (2006) The grounded theory method: Application of a variant of its procedure of constant comparative analysis to psychotherapy research. In Fischer CT (Ed.), Qualitative Research Methods for Psychologists-Introduction Through Empirical Studies. Amsterdam, The Netherlands: Elsevier Academic Press, pp. 59–78.

[bibr22-03080226251335228] Rezaei Z Jalali A Jalali R , et al. (2020) Haemodialysis patients’ experience with fatigue: A phenomenological study. British Journal of Nursing 29(12): 684–690. 32579460 10.12968/bjon.2020.29.12.684

[bibr23-03080226251335228] Thompson Burdine J Thorne S Sandhu G (2021) Interpretive description: A flexible qualitative methodology for medical education research. Medical Education 55: 336–343. 32967042 10.1111/medu.14380

[bibr24-03080226251335228] Thorne S (2016). Interpretive Description. New York, NY: Routledge.

[bibr25-03080226251335228] Tong A Manns B Hemmelgarn B , et al. (2017) Establishing core outcome domains in hemodialysis: Report of the standardized outcomes in Nephrology-Hemodialysis (SONG-HD) Consensus Workshop. American Journal of Kidney Diseases 69: 97–107. 27497527 10.1053/j.ajkd.2016.05.022 PMC5369351

